# Hygrometric Properties of Glycerine

**Published:** 1879-09

**Authors:** 


*Hygrometric Properties of Glycerine.*—The use of glycerine in many cases is indicated as an emolient, etc., but often its application to sensitive surfaces causes pain, due to its great affinity for the water of the tissues. It is interesting to know that although it attracts moisture and an uncovered vessel of glycerine will imbibe it from the air, that there is a limit to its hygrometric properties, such limit being found by careful experiment to be three parts by measure of glycerine to one of water. Beyond this, its point of saturation, it does not absorb water. When therefore the application of glycerine to an inflamed or denuded surface is found desirable, the dilution of this substance to the extent above suggested, it will be found a satisfactory means of remedying the smarting the pure article ordinarily causes. H.

